# Therapeutic Potential of hucMSC-EVs in Diabetic Kidney Disease via Regulating the miR-146b-5p/Merlin/YAP Axis

**DOI:** 10.1155/sci/5243270

**Published:** 2025-10-28

**Authors:** Bei Li, Qiongni Wang, Linru Shi, Qifeng Liu, Hui Qian, Lixia Yu, Cheng Ji

**Affiliations:** ^1^Department of Nephrology, Affiliated Kunshan Hospital of Jiangsu University, Kunshan 215300, China; ^2^Jiangsu Key Laboratory of Laboratory Medicine, Department of Laboratory Medicine, School of Medicine, Jiangsu University, Zhenjiang 212013, China; ^3^Department of Laboratory Medicine, Ningbo Hangzhou Bay Hospital, Ningbo 315336, China

**Keywords:** hucMSC-EVs, miR-146b/merlin/YAP, renal fibrosis, ubiquitination degradation

## Abstract

Diabetic kidney disease (DKD) is characterized by a continuous decline in renal function and progressive fibrosis, making it a leading cause of end-stage kidney disease with limited therapeutic options. Recently, extracellular vesicles derived from mesenchymal stem cells (MSC-EVs) have shown great potential in tissue regeneration and repair, offering a new avenue for the treatment of DKD. The purpose of this study is to explore the function and mechanism of action of EVs derived from human umbilical cord MSCs (hucMSC-EVs) in the development of DKD. Our findings show that under high-glucose (HG) conditions, miR-146b-5p is highly expressed in glomerular mesangial cells, downregulating the target protein Merlin, which promotes the activation of the YAP signaling pathway and induces mesangial cell fibrotic-like changes, leading to a significant deposition of collagen and interstitial fibrosis in the kidney. In vivo and in vitro experimental findings reveal that hucMSC-EVs can significantly inhibit miR-146b-5p, upregulate Merlin expression, prevent the nuclear translocation of YAP, improve renal function, and reduce collagen deposition, demonstrating a significant antifibrotic effect. The findings of this study emphasize the central role of the miR-146b-5p/Merlin/YAP axis in hucMSC-EVs-mediated inhibition of renal fibrosis and highlight the potential of MSC-EVs as a targeted nanotherapeutic strategy for DKD.

## 1. Introduction

Diabetic kidney disease (DKD), as a form of chronic kidney disease (CKD) caused by diabetes [1], is one of the most serious microvascular complications of diabetes [2]. Renal fibrosis, a common final pathway in all progressive kidney diseases, is characterized by the gradual accumulation of proteins such as collagen, which further exacerbates kidney damage [3, 4]. Therefore, preventing and delaying the progression of DKD is crucial for improving the survival rate and life quality of patients with diabetes. Despite the significant public health challenge posed by DKD, currently effective treatment options are limited, and treatment outcomes are not ideal [5].Thus, exploring new therapeutic strategies is of great importance for improving the prognosis of patients with DKD [6, 7].

The latest advancements in regenerative medicine have highlighted the potential of extracellular vesicles derived from mesenchymal stem cells (MSC-EVs) in treating various kidney diseases [8, 9], including DKD [10]. MSC-EVs are a class of small vesicles with a membrane structure that carry a multitude of functional biomolecules, such as proteins, lipids, and RNA, which can regulate cell behavior and contribute to tissue repair [11–13]. The unique properties of MSC-EVs, such as their ability to enhance intercellular communication and facilitate the transfer of therapeutic cargo, make them an attractive therapeutic option targeting the underlying mechanisms of DKD [14, 15].

Recent research findings have highlighted the vital role of the Hippo/YAP signaling cascade in renal development, including the embryonic development of the glomerulus and lower urinary tract, maintaining the balance of podocytes, preserving the integrity of the glomerular filtration membrane, and regulating the expansion of renal cysts [16]. Throughout the Hippo signaling pathway, the Merlin/NF2 tumor suppressor, which interacts with core kinase components, serves as a common upstream activator. Subsequently, activated Mst1/2, in conjunction with Sav1, forms a complex that phosphorylates and activates Lats1/2, which in turn phosphorylates YAP/TAZ [17, 18]. In particular, YAP has emerged as a key regulatory factor in renal tissue injury and fibrosis [18, 19]. The activation of YAP correlates with the advancement of multiple kidney diseases, including DKD [20]. Under hyperglycemic conditions, YAP signaling is often upregulated, leading to increased collagen deposition and subsequent renal fibrosis [21]. Our research team's previous studies have also shown that high-glucose (HG), mechanical stress and TGF-β1 stimulation can induce the expression of YAP in the kidney [22]. Moreover, the deletion of YAP in mesangial cells can reduce renal interstitial fibrosis [23]. These findings suggest that YAP plays a vital role in the progression of kidney diseases.

In the study, we aimed to investigate the role of EVs derived from human umbilical cord MSCs (hucMSC-EVs) in the progression of DKD. We found that HG stimulation resulted in high expression of miR-146b-5p in HBZY-1 cells, downregulation of the target protein Merlin, and inhibition of the Lats1 and MST1 proteins, which led to the intranuclear accumulation of YAP and the deposition of α-SMA during DKD progression. In summary, we confirmed that the miR-146b-5p/Merlin/YAP axis accelerates the fibrotic process, and hucMSC-EVs, by mediating the ubiquitination and degradation of YAP in interstitial cells, reduce collagen deposition. Our research results suggest that hucMSC-EVs therapy could provide a novel stem cell treatment approach for kidney repair.

## 2. Materials and Methods

### 2.1. Isolation and Characterization of hucMSC-EVs

Cultivate MSCs from passage P3 to P7 using 10% extracellular vesicles-depleted FBS, and collect the culture supernatant to obtain extracellular vesicles. Next, sequentially remove cell debris from the culture supernatant (4°C, 2000 *g*, centrifugation for 30 min), and eliminate residual organelles (4°C, 10,000 *g*, centrifugation for 30 min). Use a 100 kDa ultrafiltration tube to concentrate the supernatant obtained after 2000 *g* centrifugation for 30 min. Then perform ultracentrifugation (4°C, 100,000 *g*, for 3 h), discard the supernatant, and resuspend the pellet containing MSC-EVs in PBS. Finally, characterize and identify the obtained MSC-EVs.

### 2.2. Cell Culture

After obtaining consent from the obstetric mother at the Affiliated Hospital of Jiangsu University, the umbilical cords were collected and processed within 6 h. HucMSCs were cultured in α-MEM medium supplemented with nucleosides (Meilunbio), along with the addition of 10% fetal bovine serum (Vazyme). The HBZY-1 glomerular mesangial cell line was obtained from the Stem Cell Bank of the Chinese Academy of Sciences and maintained in DMEM medium (Meilunbio) supplemented with 10% FBS. For high osmotic pressure control, mannitol (Aladdin) was added to the culture medium at a concentration of 30 mM.

### 2.3. Animals Model

All procedures involving animals were performed following the guidelines set by the Ethics Committee of Jiangsu University (2022264, Jiangsu, China), which had granted approval for the study protocol. The male Sprague-Dawley rats, aged 8 weeks and weighing around 200 g, were procured from the Shanghai Institute for Laboratory Animals, Chinese Academy of Sciences, and maintained in the animal research facility of Jiangsu University. Male SD rats were fed a high-fat diet (45% kcal from fat) for 4 weeks, fasted overnight, and then received a single tail-vein injection of streptozotocin (STZ, 35 mg kg^−1^). Animals with fasting blood glucose ≥ 16.7 mmol L^−1^ on two consecutive measurements, accompanied by polydipsia and polyuria, were considered to have successfully developed DKD; those not meeting these criteria were excluded. hucMSC-EVs were administered at 10 mg kg^−1^ (total protein quantified by BCA) via tail-vein injection. Normal rats, maintained on standard chow for 24 weeks, served as controls. Euthanasia was performed under isoflurane anesthesia, and kidney tissues were harvested for further analysis.

### 2.4. Quantitative Reverse Transcription PCR

Following the guidelines provided by Invitrogen, total RNA was extracted from mouse kidneys and HBZY-1 cells using Trizol reagent. Subsequently, RNA was reverse-transcribed into cDNA using the HiScript III 1st cDNA Synthesis Kit (with gDNA removal) from Vazyme. qPCR experiments were conducted with the ChamQ Blue Universal SYBR qPCR Master Mix (Vazyme), and gene expression levels were determined by the 2^−ΔΔCT^ method, utilizing β-actin as the endogenous control. For the detection of miR-146b-5p, the miRNA1st Strand cDNA Synthesis Kit (stem-loop based) from Vazyme was employed, and its expression relative to U6 was determined using the 2^−ΔΔCt^ method. Each experimental group included six or three biological replicates. The PCR primers used in the experiments are listed in Table 1.

### 2.5. Analysis of GEO Dataset Information

Access the GEO database homepage (https://www.ncbi.nlm.nih.gov/geo/), retrieve the normalized gene-expression profiles of microdissected human renal biopsy tissues from dataset GEO (GSE47183), and summarize miR-146b-5p levels in *n* = 23 normal controls, *n* = 44 DKD patients, *n* = 34 FSGS patients, *n* = 30 IgAN patients, and *n* = 34 MPGN patients. Perform *t*-tests to compare miR-146b-5p expression between controls and each disease group. Apply the same workflow to GEO dataset GSE61741 to extract and summarize miR-146b-5p expression data for *n* = 94 normal controls and *n* = 15 longevity individuals, *n* = 124 Wilms tumor patients, and *n* = 20 renal cancer patients.

### 2.6. Histology and Immunohistochemistry

For histological examination to observe the composition and structure of different tissues, rat kidneys are perfused through the left ventricle, fixed with 4% paraformaldehyde (PFA), paraffin-embedded, and kidney sections are prepared. Subsequently, HE, Sirius Red, and Masson staining are performed. In addition to staining, immunohistochemical staining was performed for α-SMA (1:100, BioWorld), Merlin (1:200, CST), YAP (1:100, CST, USA), and MST1 (1:100, CST) to observe their expression and localization in different groups. In assessing the degree of injury, three tissue sections from each mouse are randomly selected for staining and evaluated according to the following semiquantitative scoring criteria: 0, no damage; 1, < 25%; 2, 25 to ~50%; 3, 50 to ~75%; 4, > 75%. Sirius Red and Masson's trichrome staining areas were semi-quantitatively assessed using the ImageJ (Fiji) software.

### 2.7. Dual-Luciferase Reporter Gene Assay

293T cells, derived from human embryonic kidney, were used in the experiment. A quantity of 2 × 10^4^ cells per well was plated in a 24-well plate and incubated for a duration of 24 h. The following transfection reagents were added to each group: 25 μM NC + 200 ng wild type (WT) plasmid, 25 μM NC + 200 ng mutant (MU) plasmid, 25 μM mimics + 200 ng WT plasmid, and 25 μM mimics + 200 ng MU plasmid. Subsequently, 1 μL of Lipofectamine 2000 was added to each well, with each group repeated three times. After 24 h of culture, the dual luciferase reporter assay system (Promega, USA) was used for detection.

### 2.8. miR-146b-5p Mimics or Inhibitors Transfection

Following the instructions provided by Polyplus (101,000,046), 1.5 × 10^5^ cells were seeded per well in a six-well culture plate. miR-146b-5p mimics (25 and 50 nM) and miR-146b-5p inhibitors (50 and 100 nM) were first mixed with jetPRIME buffer, then jetPRIME reagent was added for transient transfection into HBZY-1 cells, with the cells reaching 70%–80% confluence in the six-well culture plates. After continued culture for 48 h, total RNA and proteins were harvested for further experiments.

### 2.9. Cellular Uptake of MSC-EVs In Vitro

Take 1000 μL of MSC-EVs (with a total particle count around 10^11^), add 1 μL of Dil (Invitrogen, V22888) dye and mix well, then incubate at room temperature for 30 min. Transfer to a 100 KD ultrafiltration tube and centrifuge at 4°C, 2000 *g*, for 15 min to collect the concentrated solution. Add this to HBZY-1 cells for coculturing for 12 h. Unlabeled MSC-EVs served as the control group and were cocultured with the cells. Fix with a 4% PFA solution for 15 min, stain the cytoskeleton with phalloidin (1:1000, uelandy, P0059L) for 1 h, and stain the nucleus with hoechst33342(1:300, sigma, B2261) for 5 min. Mount the cover slip and observe and photograph under a confocal microscope (DeltaVision Elite, GE, USA).

### 2.10. Western Blot Analysis

Tissue and intracellular proteins were extracted using RIPA lysis buffer (Thermo Scientific, 89,901) containing a mixture of protease and phosphatase inhibitors Mini (Pierce, A32961). After protein extraction, the BCA assay method was used to quantitatively analyze the protein content to determine its concentration. Subsequently, equal amounts of sample lysates were subjected to electrophoretic separation on 10% or 12% SDS-PAGE gels (Vazyme). After completion of electrophoresis, the proteins were transferred onto a PVDF membrane for Western blot analysis. After blocking the membrane with a 5% milk solution for 2 h, the membrane was then incubated with the primary antibody at 4°C for the duration of the night, including CD9, CD63, CD81, Alix, TSG101, and negative control Calnexin (1:400, CST, USA), α-SMA (1:300, BioWorld, P62736), Col1a1 (1:500, BioWorld, BS70155), Merlin (1:500, CST, USA), YAP (1:300, CST, USA), Lats1 (1:500, CST), β-actin (1:2000, CWBIO, China), and GAPDH (1:2000, Abclonal, China, AC027). On the following day, the membrane was incubated with the corresponding secondary antibodies at room temperature for 2 h. Subsequently, the membrane was developed using the ECL detection system. The band density of the Western blot was analyzed using ImageJ software.

### 2.11. Immunofluorescence Staining

Kidney tissue sections (5 μm thick) were immersed in a 4% PFA solution and fixed for 12 h at 4°C. Then, they were permeabilized with phosphate-buffered saline (PBS) containing 0.15% TritonX-100 for 30 min, followed by blocking with 5% bovine serum albumin (BSA) for 1 h to prevent non-specific antibody binding. For HBZY-1 cells, fixation was first performed with 4% PFA for 15 min, followed by membrane permeabilization with a 0.2% Triton X-100 solution at room temperature for 10 min. Subsequently, the cells were blocked with a solution containing 5% BSA for 30 min. The sections were then incubated with the following primary antibodies: YAP (1:100, CST), Merlin (1:100, CST), α-SMA (1:100, CST), and incubated at 4°C for 24 h. After washing with PBS, the sections were incubated with secondary antibodies at 37°C for 45 min. The cell nuclei were subsequently stained with Hoechst33342 (1:200, Sigma) for 5 min. Ultimately, the sections were observed using a DeltaVision Elite confocal microscope.

### 2.12. Statistical Analysis

Data are presented as the mean ± SD. Statistical analysis was performed using GraphPad Prism 9.5 software, with either *t*-tests or one-way analysis of variance (ANOVA) employed. Results with a *p*-value less than 0.05 were indicated with an asterisk (*⁣*^*∗*^) to denote statistical significance. *⁣*^*∗∗*^*p* < 0.01, *⁣*^*∗∗∗*^*p* < 0.001, *⁣*^*∗∗∗∗*^*p* < 0.0001.

## 3. Results

### 3.1. The Level of miR-146b-5p Increases With the Progression of DKD

**Figure 1:**
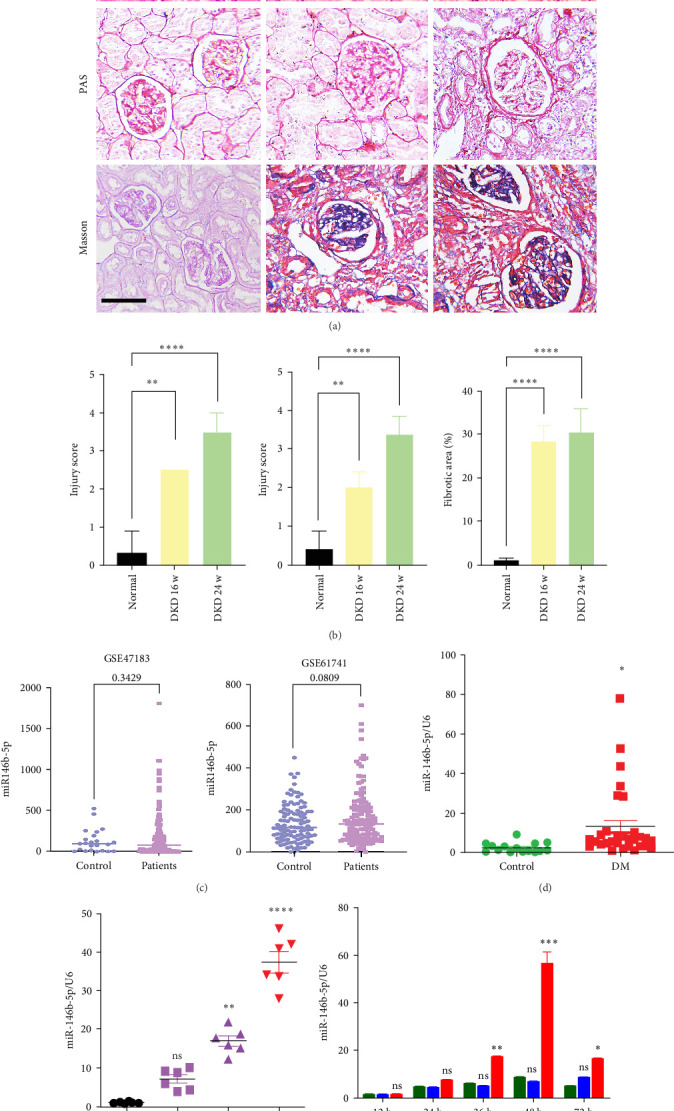
The level of miR-146b-5p increases with the progression of DKD. (A) Representative images of HE, PAS, and Masson staining of DKD renal tissues. Scale bar, 100 μm. (B) Renal injury was assessed on the basis of hematoxylin-eosin (H&E) and PAS staining, and renal fibrosis was assessed on the basis of Masson staining. (C) Comparison of miR-146b-5p levels between control and CKD patients in the GSE47183 dataset and between normal, long-lived, and renal cancer, nephroblastoma groups in the GSE61741 dataset. (D) qRT-PCR analysis of miR-146b-5p expression in the serum of diabetic patients and healthy controls. (E) qRT-PCR analysis of miR-146b-5p expression in normal and 8, 16, and 24-week DKD renal tissues. (F) qRT-PCR detection of miR-146b-5p expression in NRK-52E, podocytes, and HBZY-1 cells stimulated by high-glucose at different time points. ns, not significant; *⁣*^*∗*^*p* < 0.05; *⁣*^*∗∗*^*p* < 0.01; *⁣*^*∗∗∗*^*p* < 0.001; *⁣*^*∗∗∗∗*^*p* < 0.0001.

To investigate the mechanisms underlying renal fibrosis in DKD, we established a rat model of DKD. Through pathological assessments using HE, Sirius Red, and Masson staining, we observed a significant thickening of the glomerular basement membrane and substantial collagen deposition in the renal interstitium in the DKD rat model compared to healthy rats. (Figure 1A,B). Subsequently, by analyzing data from GSE47183 and GSE61741, we identified miR-146b-5p as a sensitive indicator of kidney disease progression, with elevated levels of miR-146b-5p observed in both CKD and kidney-related cancers (Figure 1C). Considering the results from clinical specimens, we collected serum samples from 50 diabetic patients and 40 healthy controls and found, through qRT-PCR analysis, that miR-146b-5p was highly expressed in the diabetic group (Figure 1D). Further analysis revealed that circulating miR-146b-5p levels positively correlated with serum CREA (*r* = 0.571, *p* < 0.001), BUN (*r* = 0.469, *p* < 0.001), and eGFR (*r* = 0.439, *p* < 0.001), and exhibited significant diagnostic value for DKD (Figure S1). Accordingly, we studied the temporal expression of miR-146b-5p in renal tissues over 24 weeks and found its expression to be significantly upregulated (Figure 1E). In vitro experiments corresponding to NRK-52E and podocytes revealed that miR-146b-5p was primarily predominantly expressed in HBZY-1 cells stimulated by HG (Figure 1F). The above results indicate that miR-146b-5p is a key factor in the progression of DKD.

### 3.2. The Merlin/YAP Axis is a Potential Target of miR-146b-5p in the Progression of DKD

**Figure 2:**
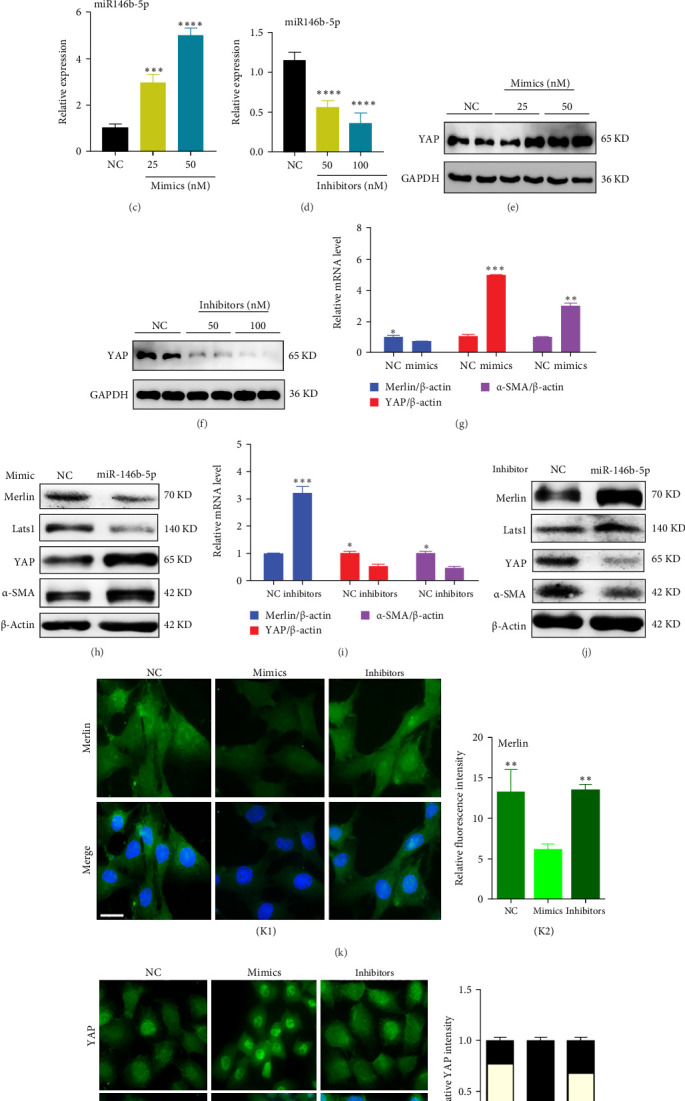
The Merlin/YAP axis is a potential target of miR-146b-5p in the progression of DKD. (A) Prediction of binding sites between NF2 3′UTR and hsa-miR-146b-5p. (B) Dual-luciferase reporter gene analysis: WT: dual-luciferase plasmids transfected with wild type NF2 3′UTR sequence; MU: dual-luciferase plasmids transfected with mutated NF2 3′UTR sequence. (C) qRT-PCR detection of miR-146b-5p expression in mesangial cells transfected with miR-146b-5p mimics. (D) qRT-PCR detection of miR-146b-5p expression in mesangial cells transfected with miR-146b-5p inhibitors. (E) Assessment of YAP protein expression levels after transfection of cells with miR-146b-5p mimics by Western blot analysis. (F) Assessment of YAP protein expression levels after transfection of cells with miR-146b-5p inhibitors by Western blot analysis. (G) qRT-PCR analysis was performed to examine the mRNA expression of genes after transfection with miR-146b-5p mimics. (H) Western blotting was employed to detect the expression of Merlin, Lats1, and YAP proteins after transfection with 50 nM miR-146b-5p mimics. (I) qRT-PCR analysis was performed to examine the mRNA expression of genes after transfection with miR-146b-5p inhibitors. (J) Western blotting was used to analyze the expression of Merlin, Lats1, and YAP proteins after transfection with 100 nM miR-146b-5p inhibitors. (K) Confocal microscopy analysis of Merlin expression after transfection with miR-146b-5p mimics and inhibitors (K1) and statistical analysis of Merlin expression (K2). Scale bars, 50 μm. (L) Analysis of YAP expression using confocal microscopy post-transfection with miR-146b-5p mimics and inhibitors (L1) and statistical analysis of YAP expression in the cytoplasm and nucleus (L2). Scale bars, 50 μm. ns, not significant; *⁣*^*∗*^*p* < 0.05; *⁣*^*∗∗*^*p* < 0.01; *⁣*^*∗∗∗*^*p* < 0.001; *⁣*^*∗∗∗∗*^*p* < 0.0001.

We used the TargetScan program to predict potential targets of miR-146b-5p and selected the NF2 gene as a target gene for study (Figure 2A). Subsequently, to demonstrate that miR-146b-5p directly regulates the NF2 gene, also known as Merlin, we inserted the 3′-UTR of Merlin's mRNA sequence into a luciferase-based reporter plasmid. The dual-luciferase assay revealed that the overexpression of miR-146b-5p led to a decrease in luciferase signal in 293T cells carrying the WT reporter construct. Conversely, this effect was not observed in cells harboring the MU version of the reporter construct (Figure 2B). To verify that miR-146b-5p can regulate the Hippo/YAP pathway by inhibiting the translation of Merlin protein, we validated its downstream pathway by transfecting miR-146b-5p mimics and inhibitors. After transfection of mimics and inhibitors in HBZY-1 cells, qRT-PCR analysis indicated that the expression of miR-146b-5p gradually increased (Figure 2C) or decreased (Figure 2D) with the transfection dose of mimics or inhibitors. Western blot analysis confirmed that, compared to their respective control groups (NC), the expression of YAP was significantly higher in the mimics group (Figure 2E), while it was markedly reduced in the inhibitors group (Figure 2F).

We then selected concentrations of 50 nM mimics and 100 nM inhibitors for transfection and observed through qRT-PCR and Western blot analysis that after transfection of miR-146b-5p mimics, the expression of Merlin was suppressed, Lats1 in the Hippo pathway was inactivated, and the expression of YAP and α-SMA increased in HBZY-1 cells (Figure 2G,H). Conversely, knockdown of miR-146b-5p restored the expression of Merlin protein and activated downstream Lats1, reducing the expression of YAP and α-SMA in HBZY-1 cells (Figure 2I,J). Using confocal microscopy, we observed the distribution of Merlin and YAP in mesangial cells after transfection with miR-146b-5p mimics or inhibitors. The results showed that the expression of Merlin in the cytoplasm decreased after transfection with mimics, while it increased after transfection with inhibitors (Figure 2K). Correspondingly, the expression of YAP in the nucleus increased after transfection with mimics but decreased after transfection with inhibitors (Figure 2L). The above results indicate that miR-146b-5p can downregulate the target protein Merlin and inhibit Lats1 in the Hippo pathway, thereby promoting nuclear expression of YAP and deposition of α-SMA, suggesting that miR-146b-5p can accelerate the fibrotic process of DKD by targeting the Merlin/YAP axis.

### 3.3. The miR-146b-5p/Merlin/YAP Axis Accelerates the Fibrotic Process in DKD

**Figure 3:**
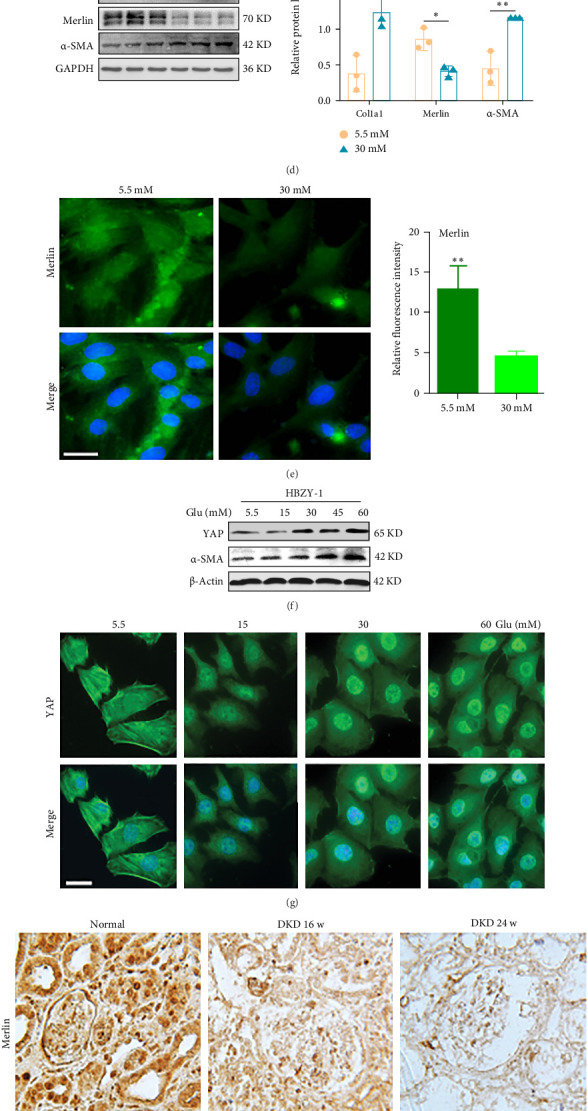
The miR-146b-5p/Merlin/YAP axis accelerates the fibrotic process in DKD. (A) Western blot analysis of Merlin in 24-week DKD kidney tissues. (B) Representative IHC images of Merlin in the 24-week DKD model. Scale bars, 100 μm. (C) Double immunofluorescence staining of Merlin (red) and α-SMA (green) in the kidneys of control and DKD rats. Scale bar, 100 μm. (D) Western blot analysis of Merlin, α-SMA, and Cola1 in HBZY-1 cells treated with high glucose. (E) Confocal microscopy analysis of Merlin in HBZY-1 cells treated with high glucose. Scale bars, 50 μm.(F) Western blot analysis of YAP and α-SMA in HBZY-1 cells treated with high glucose. (G) Confocal microscopy analysis of YAP in HBZY-1 cells treated with different concentrations of high glucose. Scale bars, 50 μm. (H) Representative IHC images of Merlin, YAP, and α-SMA in the DKD model. Scale bars, 100 μm. (I) Western blot analysis of Merlin, Lats1, YAP, and α-SMA in 24-week DKD kidney tissues. (J) qRT-PCR detection of the changes in the levels of Merlin, Lats1, YAP, and α-SMA in 24-week DKD kidney tissues. ns, not significant; *⁣*^*∗*^*p* < 0.05; *⁣*^*∗∗*^*p* < 0.01; *⁣*^*∗∗∗*^*p* < 0.001; *⁣*^*∗∗∗∗*^*p* < 0.0001.

Subsequently, we explored the specific mechanisms of the Merlin/YAP axis targeted by miR-146b-5p in the progression of DKD. In vivo, Western blot analysis of protein extracts from 24-week DKD kidney tissues showed a decrease in Merlin expression in the kidneys of 24-week DKD (Figure 3A). Immunohistochemistry and immunofluorescence of kidney tissues also revealed a reduction in Merlin expression in 24-week DKD kidney tissues (Figure 3B,C). In vitro western blot analysis results indicated that under HG conditions, the expression of Merlin protein was reduced, while the expression levels of fibrotic markers α-SMA and Collagen I were increased (Figure 3D). Confocal microscopy images revealed that in a HG environment, the expression of Merlin protein in HBZY-1 cells was significantly decreased (Figure 3E). The above results indicate that the content of Merlin decreases as DKD progresses.

Western blot analysis further revealed that HG stimulation significantly increased total YAP and the fibrotic marker α-SMA, while decreasing phosphorylated YAP (p-YAP) and p-LATS1 levels; these changes were not induced by osmotic control with mannitol (Figure S2A). Nuclear–cytoplasmic fractionation confirmed that YAP translocated from the cytoplasm to the nucleus specifically under HG, but not under hyperosmotic mannitol conditions (Figure S2B). Meanwhile, after stimulation of HBZY-1 cells with different high concentrations of glucose for 48 h, YAP was found to be highly expressed and accumulated in the nuclei of mesangial cells (Figure 3G). In vivo, representative immunohistochemical images and Western blot results demonstrated that in 24-week DKD kidney tissues, Merlin expression decreased, YAP expression in the nucleus significantly increased, and its fibrotic marker α-SMA was markedly activated (Figure 3H,I). qRT-PCR analysis of tissue protein gene levels also confirmed this phenomenon (Figure 3J). These results suggest that under hyperglycemic stress, Hippo pathway proteins Merlin and Lats1 are suppressed, YAP is activated, and when YAP translocates from the cytoplasm of renal interstitial cells to the nucleus, it stimulates the deposition of α-SMA in renal tissues, and miR-146b-5p accelerates the fibrotic process of DKD through the Merlin/YAP axis.

### 3.4. Isolation of MSCs and Characterization of Biological Properties of hucMSC-EVs

**Figure 4:**
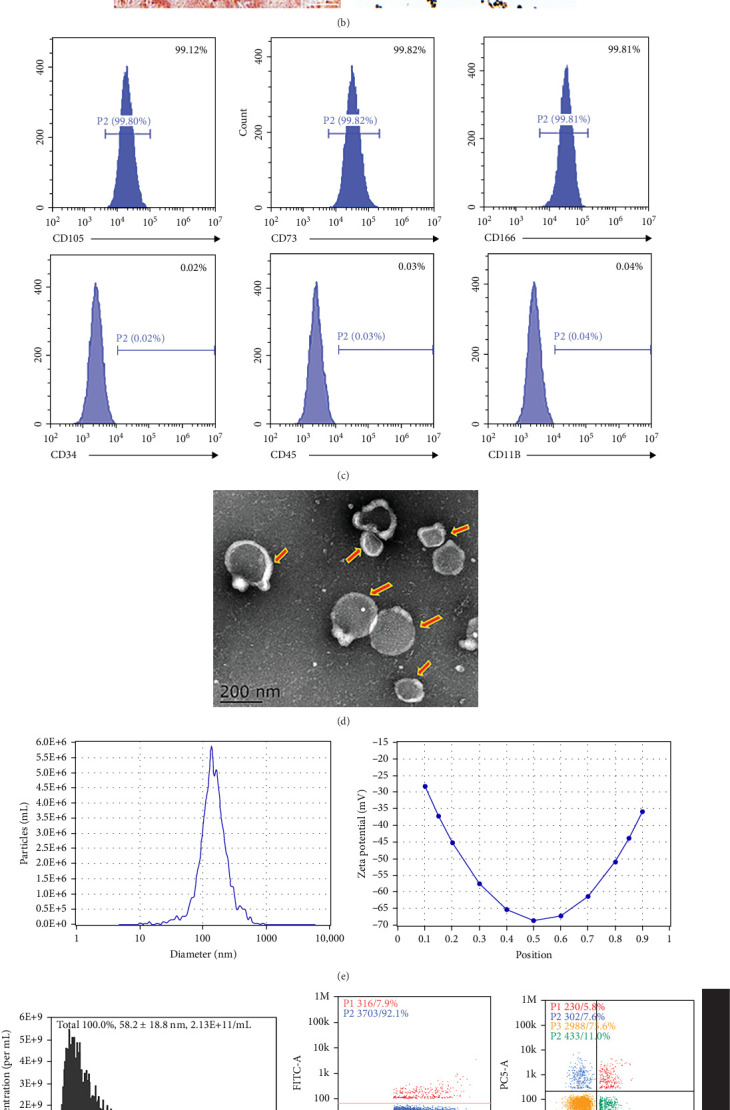
Isolation of MSCs and characterization of the biological properties of hucMSC-EVs. (A) Morphological identification of human umbilical cord tissue and primary hucMSCs ([A1], scale bars, 20 μm) and Passage 3 ([A2], scale bars, 50 μm). Scale bar, 20 μm (left) and 59 μm (right). (B) Osteogenic differentiation of hucMSCs: alizarin red S staining and adipogenic differentiation of hucMSCs: oil red o staining. Scale bar, 500 μm. (C) Analysis of the surface markers of hucMSCs via flow cytometry. (D) Representative morphology of hucMSC-Ex identified by TEM. (E) Determination of the average size and zeta potential of hucMSC-EVs using NTA. (F) Nanoflow cytometry for the size distribution of extracellular vesicles and surface markers CD81 (F1), CD9, and CD63 (F2). (G) Western blot detection of exosome positive markers CD9, CD63, CD81, TSG101, alix, and negative marker calnexin.

Human umbilical cord tissue was manually dissected, separated, and purified, and cultured in α-MEM medium containing 15% FBS for 10 days. Under the microscope, hucMSCs cells were observed to be closely arranged around the umbilical cord tissue, which are referred to as Passage 1 (P1) hucMSCs. After continuous passaging and expansion, Passage 3 (P3) hucMSCs cells were able to be stably passaged and exhibited a spindle-shaped appearance and a fish-school-like growth pattern (Figure 4A). P3 hucMSCs were subjected to osteogenic and adipogenic induction experiments. Upon microscopic examination, hucMSCs exhibited red staining with Alizarin Red S, and the presence of red lipid droplets was noted under Oil Red O staining (Figure 4B), confirming the potential of hucMSCs to differentiate into osteoblasts and adipocytes. Further analysis of hucMSC characteristics by flow cytometry showed that hucMSCs highly express typical mesenchymal stem cell surface markers CD105, CD73, and CD166, while the expression levels of CD34, CD45, and CD11B are relatively low (Figure 4C). This is consistent with the findings from other studies that have reported the expression of these markers on hucMSCs. These experiments confirmed that the hucMSCs isolated by our standardized method have high purity, strong proliferation capacity, and stable passage ability, providing a foundation for subsequent experiments. Subsequently, we extracted hucMSC-EVs from the culture supernatant of hucMSCs using ultrafiltration combined with differential ultracentrifugation. We then conducted overall characterization (protein markers) and individual characterization (transmission electron microscopy (TEM) and NTA) experiments on hucMSC-EVs. As nanoscale vesicles, extracellular vesicles were observed under TEM, exhibiting a cup-shaped spherical structure (indicated by red arrows) (Figure 4D). Verification of MSC-EVs size distribution was conducted using NTA and nanoflow cytometry techniques (Figure 4E.F). Western blot results showed that hucMSC-EVs express exosome markers (CD9, CD63, CD81, TSG101, and Alix) and do not express the endoplasmic reticulum membrane marker calnexin (Figure 4G). These data indicate that a stable and good source of hucMSCs cells is essential to ensure high bioactivity and high purity of hucMSC-EVs. Our ability to extract high-quality hucMSC-EVs is conducive to the smooth progress of subsequent experiments.

### 3.5. MSC-EVs Downregulates miR-146b-5p and Modulates Merlin/YAP Axis to Improve Fibrosis in DKD Rats

**Figure 5:**
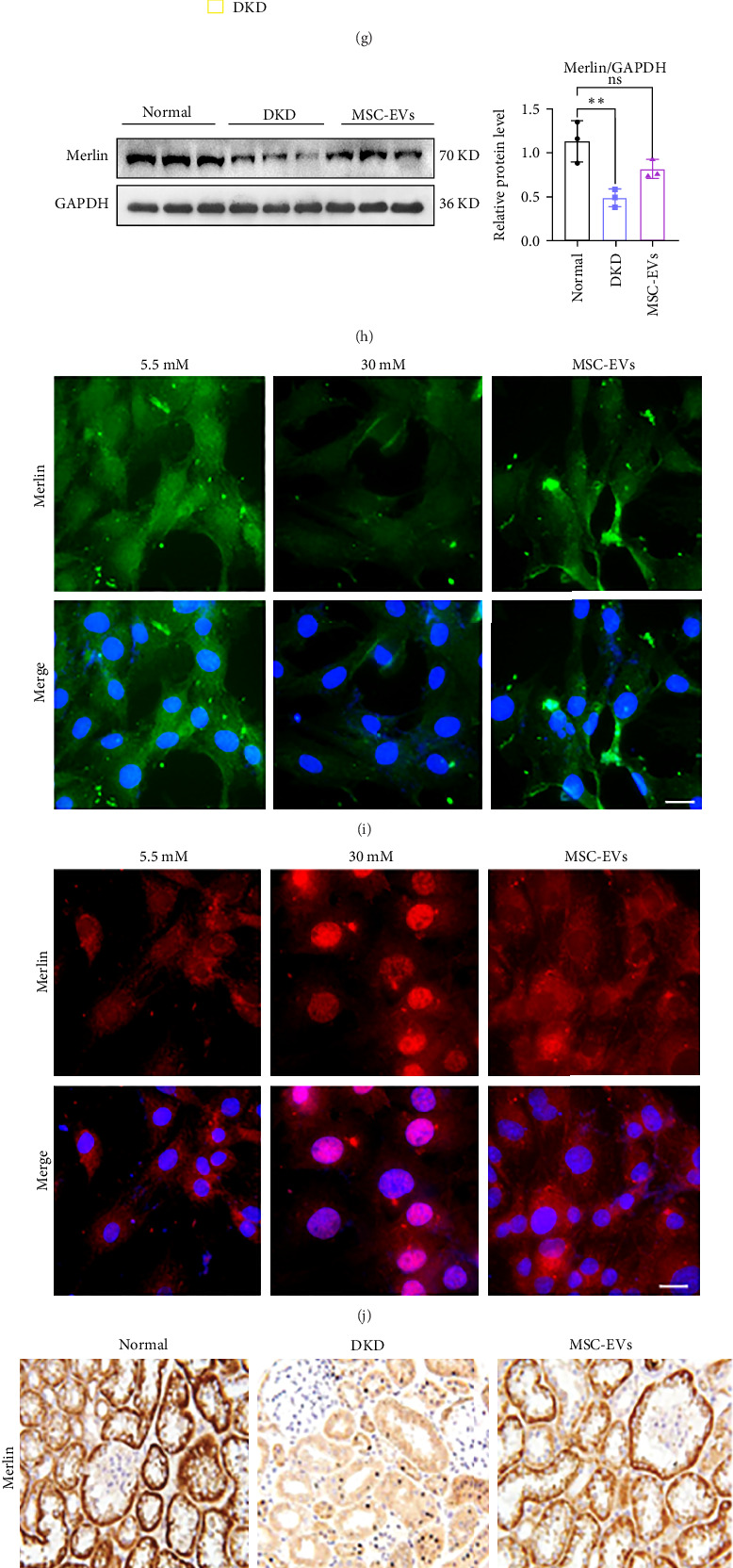
MSC-EVs restore renal function and ameliorate fibrosis in DKD rats by downregulating miR-146b-5p/Merlin/YAP axis. (A) H&E staining, Masson's staining, and sirius red staining of rat kidney tissues. Scale bar, 100 μm. (B) Dil (red) labeled MSC-EVs and phalloidin (green) labeled cytoskeleton to detect the uptake of MSC-EVs in HBZY-1 cells. Scale bars, 10 μm.(C) qRT-PCR detection of miR-146b-5p expression among various groups in the in vitro model. (D) qRT-PCR detection of Merlin, YAP, and α-SMA expression among various groups in the in vitro model. (E) Western blot analysis of Cola1, Merlin, and α-SMA in HG and MSC-EVs treated mesangial cells. (F) qRT-PCR detection of miR-146b-5p expression among various groups in the in vivo model. (G) qRT-PCR detection of Merlin, YAP, and α-SMA expression in kidney tissues from various groups in the in vivo model. (H) Western blot analysis of Merlin in kidney tissues from various groups in the in vivo model. (I) Confocal detection of Merlin expression among HG and MSC-EVs treated mesangial cells in various groups. Scale bars, 20 μm. (J) Confocal detection of YAP expression among HG and MSC-EVs treated mesangial cells in various groups. Scale bars, 20 μm. (K) Immunohistochemical staining to detect the expression of Merlin, YAP, MST1, and α-SMA in kidney tissues from various groups. Scale bar, 100 μm. (L) Tissue immunofluorescence detection of Merlin and α-SMA expression in kidney tissues from various groups. Scale bar, 100 μm. ns, not significant; *⁣*^*∗*^*p* < 0.05; *⁣*^*∗∗*^*p* < 0.01; *⁣*^*∗∗∗*^*p* < 0.001; *⁣*^*∗∗∗∗*^*p* < 0.0001.

To evaluate the therapeutic effects of MSC-EVs, we established a DKD rat model and administered MSC-EVs (10 mg/kg) intravenously every 3 days for 6 months following streptozotocin (STZ, 35 mg/kg)-induced injury. Histological analysis of H&E, Masson, and Sirius red staining revealed that MSC-EVs treatment significantly improved renal tubulointerstitial injury, thickening of the glomerular basement membrane, and extensive collagen fiber deposition (Figure 5A). Moreover, hucMSC-EV treatment markedly attenuated renal injury, as evidenced by significant reductions in serum creatinine (CREA) and blood urea nitrogen (BUN) relative to the DKD model group (Figure S3). Safety profiling revealed no significant differences in serum ALT or AST levels between the control and hucMSC-EVs groups (Figure S4A). Histological examination (H&E staining) of the heart, liver, spleen, and lungs showed no discernible pathological abnormalities in hucMSC-EV–treated mice compared with controls (Figure S4B). MSC-EVs labeled with Dil and stained with phalloidin for the cytoskeleton showed that the red fluorescence was mainly distributed in the perinuclear region, indicating that Dil-labeled MSC-EVs were successfully taken up by HBZY-1 cells (Figure 5B). Fluorescence was virtually absent in the unlabeled-EV control group, demonstrating the specificity of uptake (Figure S5).

Furthermore, we validated the reparative effects of MSC-EVs through the miR-146b-5p/Merlin/YAP signaling axis in both the DKD rat model and HG-stimulated cellular models. Initially, we detected the expression differences of miR-146b-5p among groups to verify the impact of MSC-EVs on miR-146b-5p. qRT-PCR results showed that the expression of miR-146b-5p was significantly elevated in the DKD and HG-stimulated groups, while hucMSC-EVs intervention significantly reduced its expression (Figure 5C,F), indicating that MSC-EVs have an inhibitory effect on the upregulation of miR-146b in DKD. Here, we elucidate the mechanism by which circRNA within hucMSC-EVs acts as a sponge to downregulate miR-146b-5p. We preliminarily identified and validated that circ-0002940 is enriched in hucMSC-EVs and has a targeting relationship with miR-146b-5p (Figure S6). This suggests that hucMSC-EVs regulate the Merlin/YAP signaling pathway by targeting miR-146b-5p through circ-0002940.

**Figure 6:**
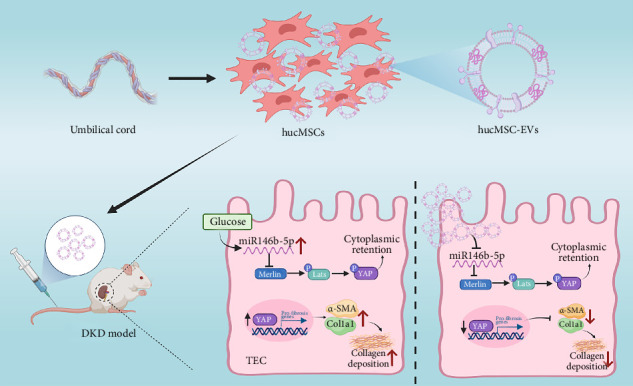
hucMSC-EVs attenuated DKD fibrosis via regulate miR-146b-5p/Merlin/YAP axis. During the progression of DKD, persistent hyperglycemia stimulates an increase in miR-146b-5p levels, downregulating the target protein Merlin and inhibiting Lats1 and MST1 proteins, thereby inducing the nuclear expression of YAP and the deposition of α-SMA in DKD renal tissue, which accelerates renal interstitial fibrosis. HucMSC-EVs, on the other hand, can suppress the content of miR-146b-5p in DKD renal tissue, restore the expression of its target protein Merlin, inhibit the nuclear entry of YAP, and alleviate renal fibrosis.

Concurrently, qRT-PCR and Western blot assays demonstrated that in both in vivo and in vitro models, Merlin protein expression decreased, while YAP protein expression increased, and fibrotic marker α-SMA protein expression was elevated; the MSC-EVs treatment group showed increased Merlin protein expression and relatively decreased YAP and α-SMA protein expression (Figure 5D,E,G,H). Confocal immunofluorescence images showed increased Merlin content after MSC-EVs treatment (Figure 5I), and MSC-EVs suppressed the high expression of YAP in the cell nucleus (Figure 5J). Additionally, immunohistochemical results indicated that MSC-EVs increased the levels of Merlin and MST1 in renal tissue and suppressed the expression of YAP and α-SMA (Figure 5K). In vivo renal tissue immunofluorescence staining showed that after hucMSC-EVs treatment, the diffuse nephropathy rat kidneys had increased cytoplasmic Merlin content, decreased α-SMA expression, and reduced colocalization with Merlin (Figure 5L). This suggests that hucMSC-EVs can ameliorate renal fibrosis in DKD by inhibiting the function of miR-146b-5p and regulating the Merlin/YAP signaling pathway in both in vivo and in vitro DKD models (Figure 6).

## 4. Discussion

This study delved into the therapeutic potential of hucMSC-EVs in DKD, with a particular focus on their ability to target the miR-146b-5p/Merlin/YAP axis to inhibit renal fibrosis. Renal fibrosis, a common pathological process in various CKDs CKD, is one of the progressive characteristics of DKD [24, 25]. Therefore, uncovering the underlying mechanisms of renal fibrosis in DKD and exploring new therapeutic strategies is of significant importance [26]. Our findings indicate that during the development of DKD, sustained hyperglycemia leads to a time-dependent increase in miR-146b-5p expression, corresponding to the downregulation of Merlin and subsequent activation of YAP. This discovery aligns with previous research emphasizing the role of noncoding RNA in disease progression [27], especially in diabetic complications [28, 29]. miR-146b-5p is upregulated in response to hyperglycemic conditions and may function in fibrotic responses by inhibiting Merlin, a negative regulator of YAP. This novel finding provides a deeper understanding of the molecular basis of DKD.

In recent years, stem cells, particularly MSCs, have garnered increasing attention in regenerative medicine. MSCs possess advantages such as pluripotency, low immunogenicity, high proliferative capacity, easy accessibility, and ethical acceptability, facilitating their clinical application [30]. It is known that MSC-EVs carry bioactive molecules, including proteins, lipids, and RNA, which can modulate cellular responses and promote tissue repair [31]. Moreover, a wealth of studies has demonstrated the immense potential of MSC-EVs in the diagnosis and treatment of kidney diseases [15, 32]. For instance, using AIEgen technology for in vivo imaging of MSC-EVs in an acute kidney injury (AKI) mouse model, it was found that MSC-EVs could specifically accumulate in the damaged kidney area and exert antioxidant effects by delivering miRNA200a–3p, thereby activating the Keap1-Nrf2 signaling axis within renal tubular epithelial cells (TECs), and promoting the repair of kidney function by improving the structure and function of mitochondria [33]. In another study, researchers discovered through miRNA sequencing analysis that MSC-EVs could ameliorate renal fibrosis by diminishing glycolysis in TECs mediated by miR-21a–5p targeting PFKM [34].

Proteomic analysis further suggests that hucMSC-EVs are more effective in tissue injury repair than exosomes derived from bone marrow MSCs (BMSCs) and adipose tissue-derived MSCs (ADSCs) [35]. Against this backdrop, our research concentrates on exploring the therapeutic effects of hucMSC-EVs in reducing kidney fibrosis linked to DKD. In our research, by intravenously injecting hucMSC-EVs into a rat model of DKD, the experimental results clearly demonstrated that hucMSC-EVs effectively halted the progression of renal fibrosis, providing strong evidence for their therapeutic potential in DKD.

In our study, we observed that MSC-EVs can effectively suppress the expression of miR-146b-5p, a microRNA that is upregulated in DKD. We further demonstrated that miR-146b-5p targets the 3′UTR of the NF2 gene of the Merlin protein, and this interaction is crucial since Merlin is known to negatively regulate YAP. Merlin primarily functions by promoting YAP phosphorylation and is an effector molecule of the Hippo signaling pathway [36]. YAP is a transcriptional coactivator that promotes cell proliferation, differentiation, and angiogenesis; it possesses only a single transcriptional activation domain and lacks a DNA-binding domain [23]. Under normal physiological conditions, p-YAP binds to 14-3-3 regulatory proteins, retaining it in the cytoplasm and modulating the expression of target factors such as Cyclin E, DIAP1, and CTGF [37]. When the Hippo pathway is blocked or inhibited, unphosphorylated YAP translocates from the cytoplasm to the nucleus, where it collaborates with TEAD to activate the overexpression of these target genes, mediating cell proliferation and extracellular matrix synthesis [37]. Our results indicate that hucMSC-EVs play a key role in promoting YAP ubiquitination and degradation miR-146b-5p, enhancing Merlin stability, and promoting its expression, effectively combating hyperglycemia-induced fibrotic signaling, reducing collagen deposition, and renal fibrosis. This underscores the potential of hucMSC-EVs as targeted nanotherapies for DKD, offering a new approach to alleviate renal fibrosis and improve patient outcomes.

Although promising results were observed in this study, it is acknowledged that there are some limitations. The long-term effects of hucMSC-EVs on renal function and fibrosis require further investigation, especially in clinical settings. Moreover, while our data indicate clear mechanistic pathways, the complexity of DKD pathology may involve other factors not explored in this study. Future research should aim to elucidate these additional factors and optimize the dosage and administration frequency of hucMSC-EVs for maximum therapeutic benefit.

In conclusion, our study provides compelling evidence supporting the therapeutic potential of hucMSC-EVs in the treatment of DKD. By targeting the miR-146b-5p/Merlin/YAP axis, hucMSC-EVs effectively alleviate renal fibrosis and improve renal function. These findings emphasize the importance of exploring stem cell-derived EVs as a novel therapeutic strategy for DKD and highlight the necessity for further research to optimize their clinical application.

## Figures and Tables

**Table 1:** The primer sequences of genes.

Gene name	Forward (5′to 3′)	Reverse (5′to 3′)
miR-146b-5p	GACGCAAAGTGCTTACAGTG	TGCAGGGTCCGAGGTAT
Merlin	CCTTGCTGCTCTACCTCCAC	ATCTGCATGGTGATGTTGGA
YAP	AAGGCTTGACCCTCGTTT	CTAATTCCCGCTCTGACG
α-SMA	CTGACTGAGCGTGGCTATTC	CCACCGATCCAGACAGAGTA
β-actin	GACCTGTACGCCAACACAGT	CTCAGGAGGAGCAATGATCT
U6	CTCGCTTCGGCAGCACA	AACGCTTCACGAATTTGC

## Data Availability

All data needed to evaluate the conclusions in the paper are present in the paper. Additional data related to this paper may be requested from the authors.

## Supporting Information

Supporting Information Figure S1. Clinical relevance of miR-146b-5p in DKD patients. Figure S2. High glucose, rather than hyperosmotic mannitol, exerts a regulatory effect on the Hippo signaling pathway. Figure S3. Functional improvement by hucMSC-EVs. Figure S4. Safety assessment of hucMSC-EVs treatment. Figure S5. Specificity of MSC-EVs uptake. Figure S6. hucMSC-EVs delivered circ-0002940 to regulate miR-146b-5p.
