# Supplementary material for: Therapeutic Potential of hucMSC-EVs in Diabetic Kidney Disease via Regulating the miR-146b-5p/Merlin/YAP Axis
Source: Stem Cells Int. 2025 Oct 28;2025:5243270. doi: 10.1155/sci/5243270 (PMC12585848; doi:10.1155/sci/5243270)
Supplement: Supporting Information — Figure S1. Clinical relevance of miR-146b-5p in DKD patients. Figure S2. High glucose, rather than hyperosmotic mannitol, exerts a regulatory effect on the Hippo signaling pathway. Figure S3. Functional improvement by hucMSC-EVs. Figure S4. Safety assessment of hucMSC-EVs treatment. Figure S5. Specificity of MSC-EVs uptake. Figure S6. hucMSC-EVs delivered circ-0002940 to regulate miR-146b-5p. [file 5243270.f1.docx]

Supplementary Materials

**Therapeutic potential of hucMSC-EVs in diabetic kidney disease**

**via regulate the miR-146b-5p/Merlin/YAP axis**

Bei Li^1,2 #^, Qiongni Wang^3 #^, Linru Shi^2^, Qifeng Liu^1^, Hui Qian^1,2*^, Lixia Yu^1*^, Cheng Ji^1,2*^

^1^ Department of Nephrology, Affiliated Kunshan Hospital of Jiangsu University, 215300, Kunshan, China.

^2^ Jiangsu Key Laboratory of Laboratory Medicine, Department of laboratory Medicine, School of Medicine, Jiangsu University, 212013, Zhenjiang, China.

^3^ Department of Laboratory Medicine, Ningbo Hangzhou Bay Hospital，315336, Ningbo, China.

* Correspondences should be addressed to:

Professor Lixia Yu, Affiliated Kunshan Hospital of Jiangsu University, 215300, Kunshan, China. E-mail: xiaoyu21@hotmail.com

DR. Cheng Ji, Affiliated Kunshan Hospital of Jiangsu University, 212002, Zhenjiang, China. E-mail: 18252586810@163.com.

# These authors contributed equally to this work


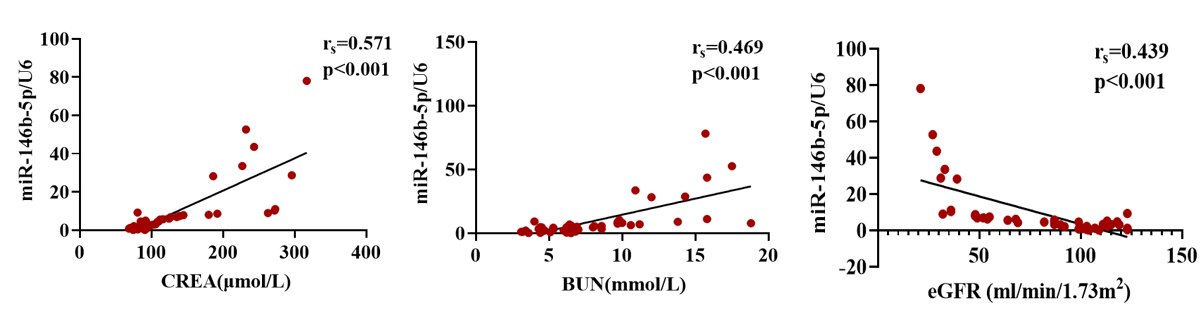


Figure S1. Clinical relevance of miR-146b-5p in DKD patients. Receiver-operating characteristic (ROC) curve analysis was employed to evaluate the diagnostic performance of circulating miR-146b-5p in discriminating DKD patients from diabetic individuals without kidney disease. Further correlation analysis revealed that miR-146b-5p levels were positively associated with serum creatinine (CREA; r=0.571, p<0.001), blood urea nitrogen (BUN; r=0.469, p<0.001), and estimated glomerular filtration rate (eGFR; r=0.439, p<0.001), underscoring its significant diagnostic value for DKD.


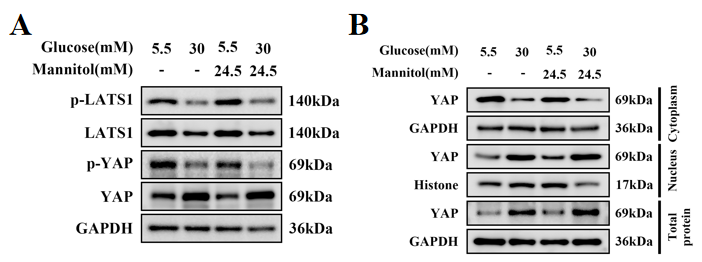


Figure S2. **High glucose, rather than hyperosmotic mannitol, exerts a regulatory effect on the Hippo signaling pathway.** (A) Western Blot analysis of p-LATS1, LATS1, p-YAP and YAP in HG and Mann treated mesangial cells. (B) HBZY-1 cells were subjected to nuclear-cytoplasmic fractionation, followed by Western blot detection of YAP after treatment with high glucose (HG) or mannitol (Mann). Histone H3 and GAPDH served as nuclear and cytoplasmic markers, respectively.


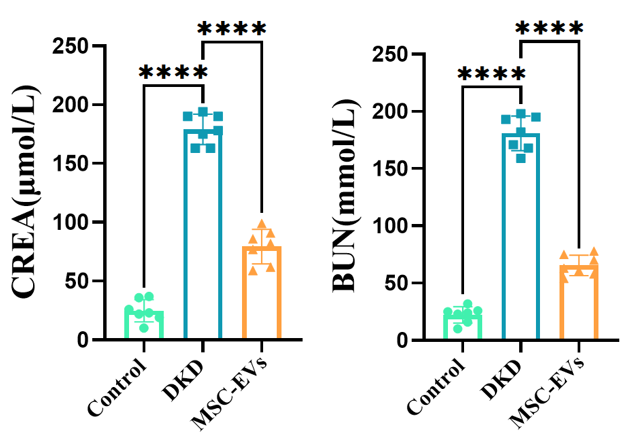


Figure S3. Functional improvement by hucMSC-EVs. Serum creatinine (CREA) and blood urea nitrogen (BUN) levels in Control, DKD, and MSC-EVs treated groups.


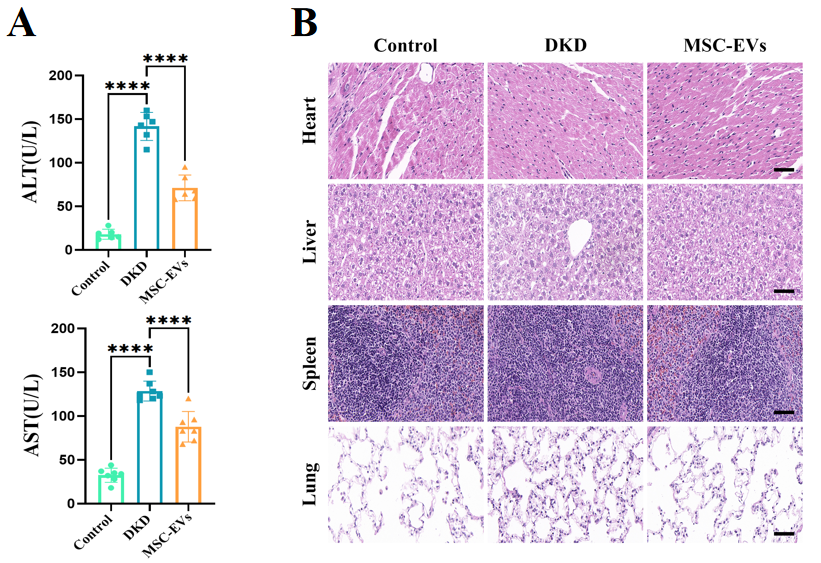


Figure S4. Safety assessment of hucMSC-EVs treatment. (A) Serum levels of alanine aminotransferase (ALT) and aspartate aminotransferase (AST) in Control, DKD, and hucMSC-EVs treated groups. (B) Representative H&E staining of heart, liver, spleen, and lung tissues from Control, DKD, and hucMSC-EVs treated groups. Scale bar, 100 μm.


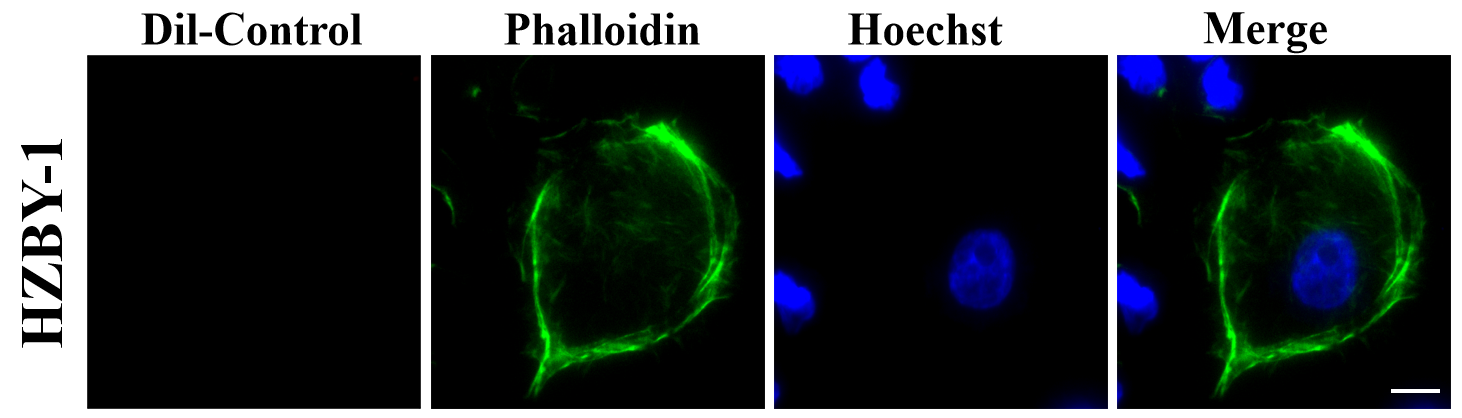


**Figure S5. Specificity of MSC-EVs uptake.** HBZY-1 cells were incubated directly with unlabeled MSC-EVs. Scale bar, 10 µm.


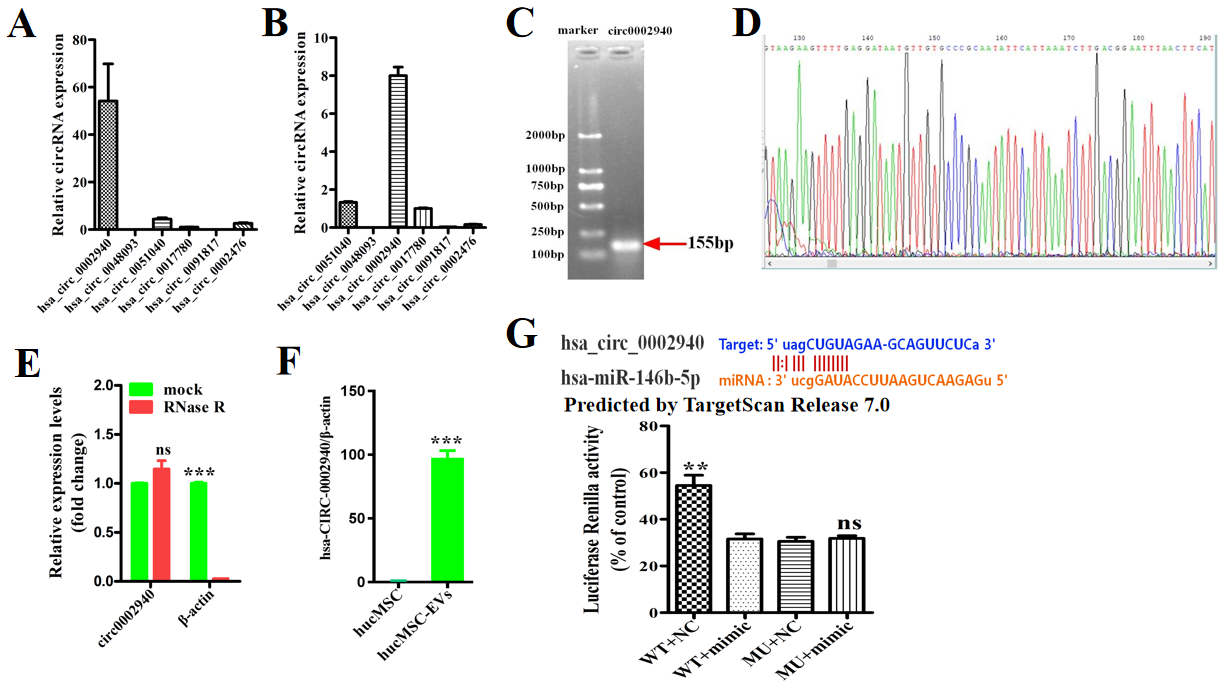


**Fig S6. hucMSC-EVs delivered circ-0002940 to regulate miR-146b-5p.** (A) qRT-PCR to detect circRNA differential expression in hucMSC; (B) qRT-PCR to detect circRNA differential expression in hucMSC-EVs; (C) agarose gel electrophoresis of the product of circ-0002940; (D) sequencing results of the product of hsa_circ_0002940; (E) RNase R resistance assay. Detect the relative expression levels of circ-0002940 and β-actin in the control (mock) and RNase R-treated groups. (F) qRT-PCR assay comparing hsa_circ_0002940 expression in hucMSC and hucMSC-EVs; (G) hsa_circ_0002940 and hsa-miR-146b-5p binding site prediction. Simultaneous dual-luciferase reporter gene analysis: WT: dual-luciferase plasmid transfected with wild-type hsa_circ_0002940 sequence; MU: dual-luciferase plasmid transfected with mutant hsa_circ_0002940 sequence (**P < 0.01).

Through StarBaseV2.0 screening of upstream molecules for miR-146b-5p, we identified six circRNAs with higher predicted binding scores to miR-146b-5p. Primers were designed and qRT-PCR analysis was performed in hucMSCs and hucMSC-EVs to select the most highly expressed molecule for subsequent experiments. qRT-PCR results demonstrated that circ-0002940 exhibited the highest expression levels in both hucMSCs (Fig. S6A) and hucMSC-EVs (Fig. S6B), indicating its enrichment in hucMSC-EVs. To confirm the circular characteristics of circ-0002940, we conducted agarose gel electrophoresis and sequencing of its product. Electrophoresis revealed a specific band of approximately 155 bp (Fig. S6C), while sequencing results confirmed the presence of a back-splice junction, thereby verifying the circular RNA nature of circ-0002940 (Fig. S6D).

To further validate the stability of circ-0002940, we evaluated its resistance to RNase R. Compared to the mock control, circ-0002940 expression decreased after RNase R treatment, while the linear RNA control β-actin exhibited a relatively smaller reduction in expression (Fig. S6E). This result highlights the relative resistance of circ-0002940 to RNase R, reinforcing its circular structure. qRT-PCR analysis of hsa_circ_0002940 expression in hucMSCs and hucMSC-EVs further confirmed its enrichment in hucMSC-EVs (Fig. S6F). Bioinformatics analysis using TargetScan Release predicted binding sites between hsa_circ_0002940 and hsa-miR-146b-5p. To verify this interaction, a dual-luciferase reporter assay was performed. Results showed that the luciferase activity of the wild-type (WT) hsa_circ_0002940 reporter was significantly suppressed by hsa-miR-146b-5p mimic, whereas no such effect was observed for the mutant (MU) reporter (Fig. S6G), confirming the direct binding of hsa-miR-146b-5p to hsa_circ_0002940.
